# Modified and dynamic intraoperativecholangiography during laparoscopic cholecystectomy in two patients with aberrant right posterior hepatic duct

**DOI:** 10.1016/j.radcr.2022.03.031

**Published:** 2022-04-04

**Authors:** Fumio Chikamori, Koji Ueta, Jun Iwabu, Niranjan Sharma

**Affiliations:** a Department of Surgery, Japanese Red Cross Kochi Hospital, 1-4-63-11 Hadaminamimachi, Kochi, 780-8562, Japan; b Adv Train Gastroint & Organ Transp Surgery, Dunedin, New Zealand

**Keywords:** Intraoperative cholangiography, Laparoscopic cholecystectomy, Aberrant right posterior hepatic duct, Bile duct injury, MRCP, DIC-CT

## Abstract

Aberrant right posterior hepatic duct (ARPHD) is one of the anatomical anomalies of the bile duct. It is a risk factor for bile duct injury during laparoscopic cholecystectomy (LC). ARPHD can be diagnosed before surgery by magnetic resonance cholangiopancreatography or drip infusion cholangiographic-computed tomography. However, it is not easy to identify ARPHD during LC. Classic intraoperative cholangiography (IOC) procedure that does not lead to bile duct injury avoidance needs to be modified. In modified IOC, cannulation is performed from the infundibulum or neck of the gallbladder. We reported a modified and dynamic IOC procedure that can identify ARPHD safely and precisely during LC. The modified IOC provided direct evidence of no injury to ARPHD in 2 cases.

## Introduction

Aberrant right posterior hepatic duct (ARPHD) is one of the anatomical anomalies of the bile duct [1], [2], [3], [4], [5]. It is a risk factor for bile duct injury during laparoscopic cholecystectomy (LC) [6], [7], [8], [9]. With the development of magnetic resonance cholangiopancreatography (MRCP) or drip infusion cholangiographic-computed tomography (DIC-CT), ARPHD can be diagnosed before surgery [10], [11], [12], [13], [14]. However, it is not easy to identify ARPHD during LC. Here we report a modified and dynamic intraoperative cholangiography (IOC) that can identify ARPHD safely during LC.

## Case 1

**Fig. 1:**
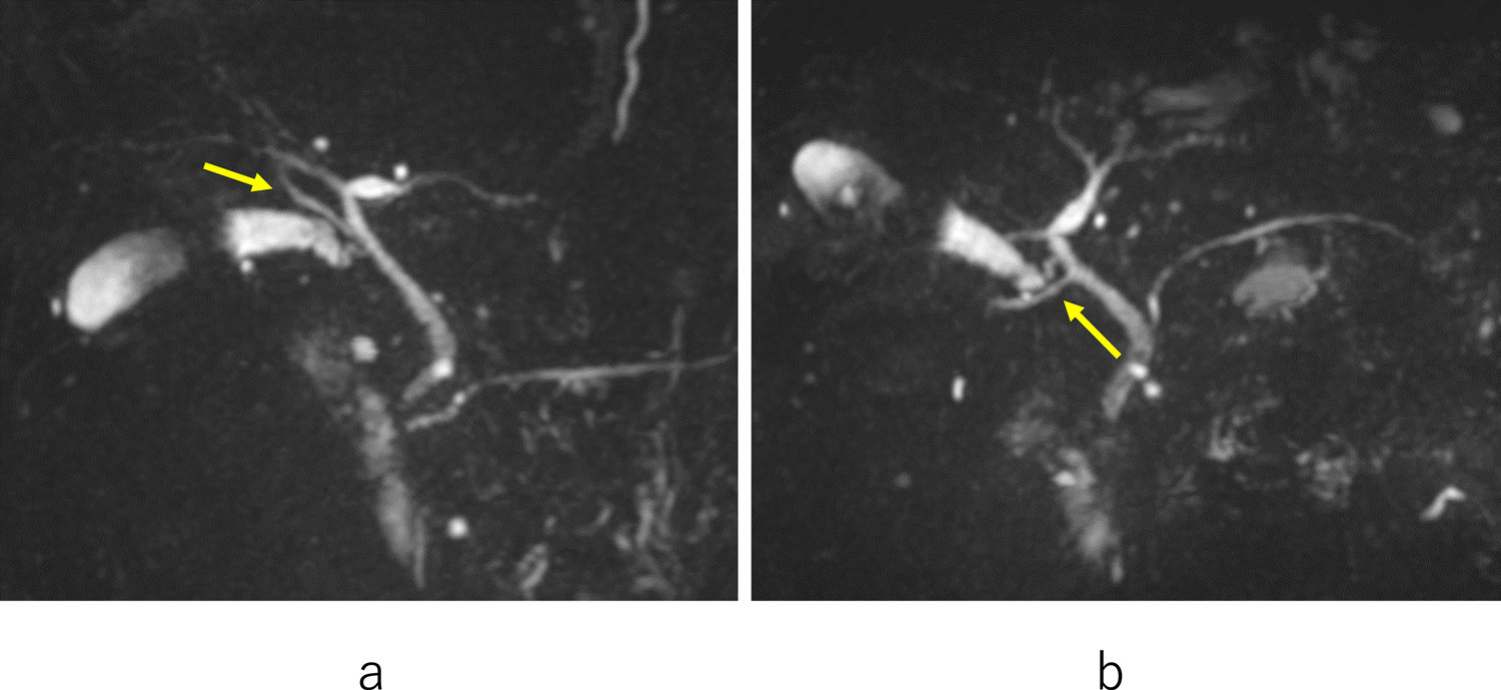
Anteroposterior view (A) and caudal view (B) of MRCP in case 1 show ARPHD draining into the extrahepatic bile duct (arrow). ARPHD, aberrant right posterior hepatic duct; MRCP, magnetic resonance cholangiopancreatography.

**Fig. 2:**
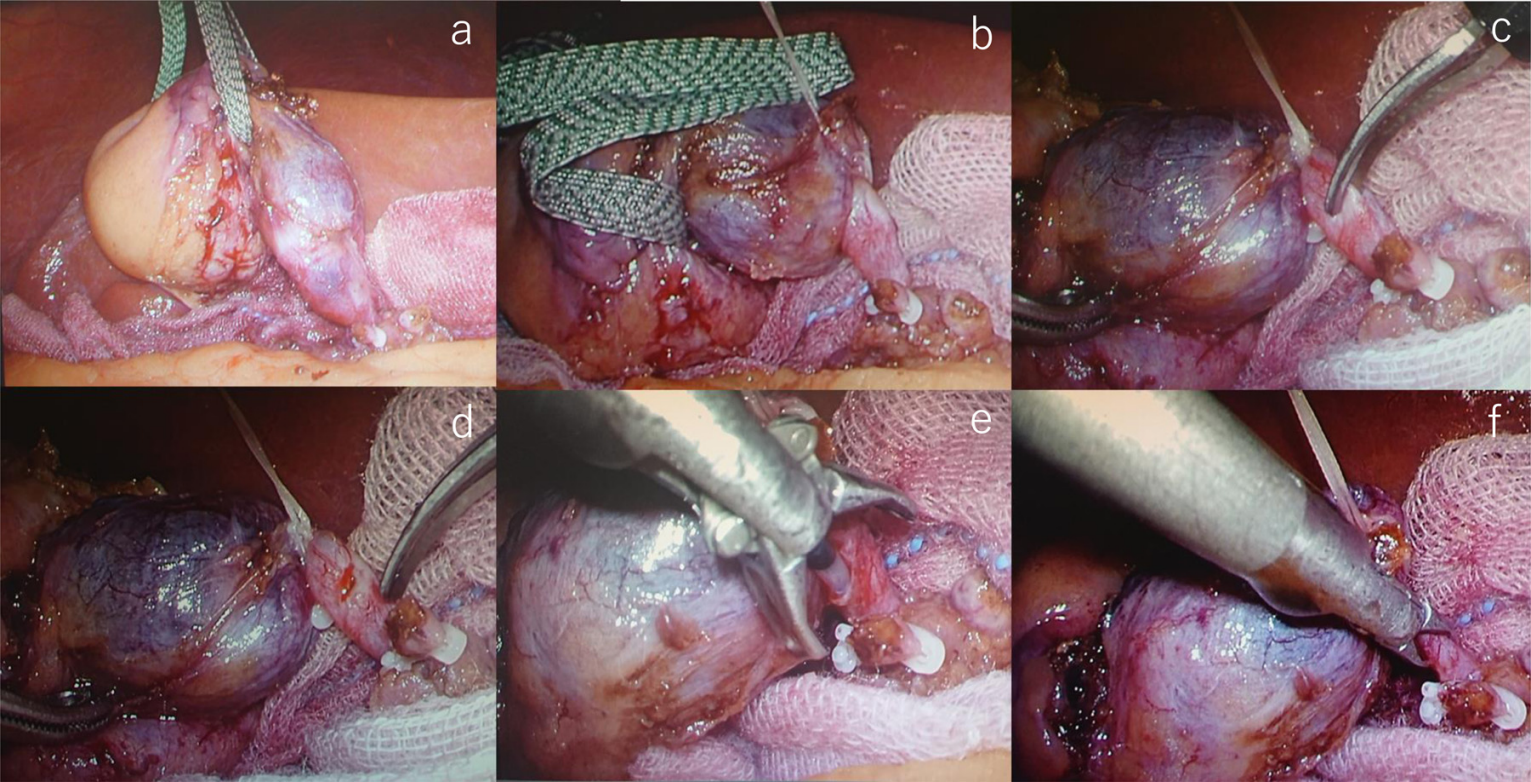
Laparoscopic view in case 1 shows the infundibulum of the gallbladder (A) that is ligated and pulled up (B). The laparoscopic view shows a small incision made at the infundibulum (C, D) and an inserted cannula for cholangiography (E, F).

**Fig. 3:**
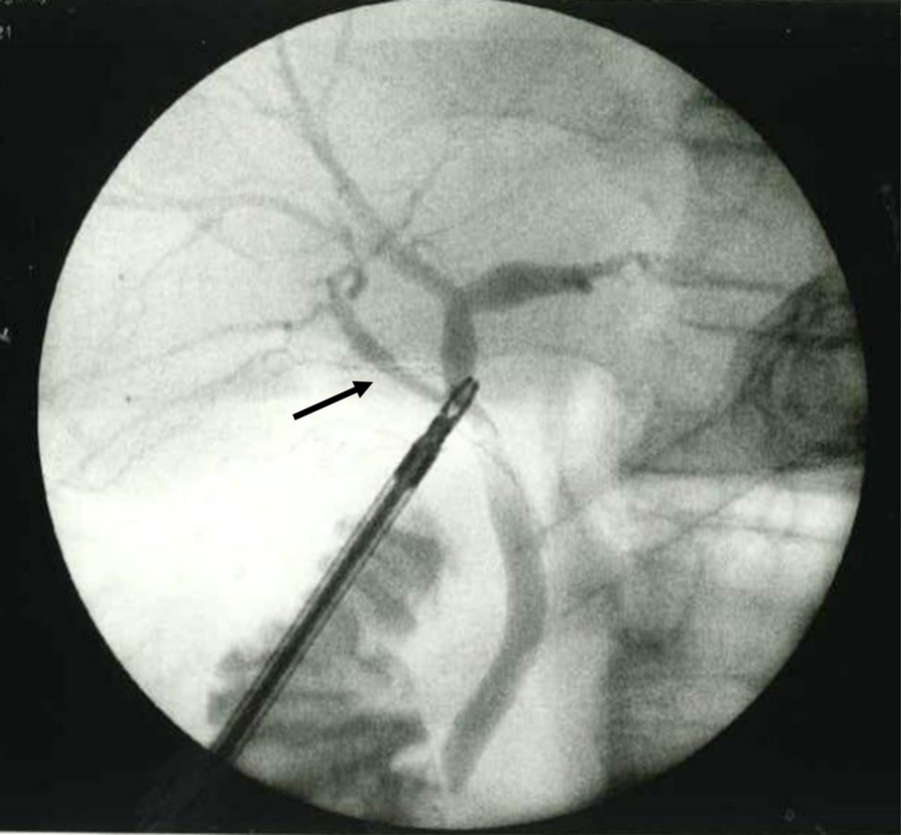
Modified IOC in case 1 shows no injury to ARPHD and no bile duct stone. ARPHD, aberrant right posterior hepatic duct; IOC, intraoperative cholangiography.

A 56-year-old male with epigastric pain was referred to our hospital for LC. Laboratory data showed no abnormalities. Abdominal ultrasonography (US) and computed tomography (CT) revealed multiple stones in the gallbladder. MRCP showed ARPHD draining into the extrahepatic bile duct (Fig. 1A, B).We diagnosed him with cholecystolithiasis and ARPHD. A 4-port LC was performed. Laparoscopy revealed mild inflammatory changes of the gallbladder. The segment IV diagonal-line approach [15] was performed and was dissected along with the inner layer of the subserosal layer (ss-i) of the gallbladder. Complete circumferential dissection of the infundibulum was achieved. Further dissection between the cystic duct and cystic artery allowed clear visualization of the cystic duct. The cystic artery was divided after one clipping. The infundibulum of the gallbladder was ligated and pulled up (Fig. 2A, B). A small incision was made at the infundibulum and a cannula for cholangiography was inserted (Fig. 2C-F). The fluoroscopic examination was performed using a mobile C-arm image intensifier. After confirming that there was no injury to ARPHD and no bile duct stone (Fig. 3), the cystic duct was doubly clipped and separated. Then the attached gallbladder was dissected from the liver bed and extracted. The patient's postoperative course was uneventful and he was discharged 2 days after LC.

## Case 2

A 74-year-old female was referred to our hospital for laparoscopic cholecystectomy. She had undergone endoscopic sphincterotomy (EST) for cholecysto-choledocholithiasis and cholangitis 1 year ago. Laboratory data showed no abnormalities. Abdominal US and CT revealed multiple small stones in the gallbladder.

**Fig. 4:**
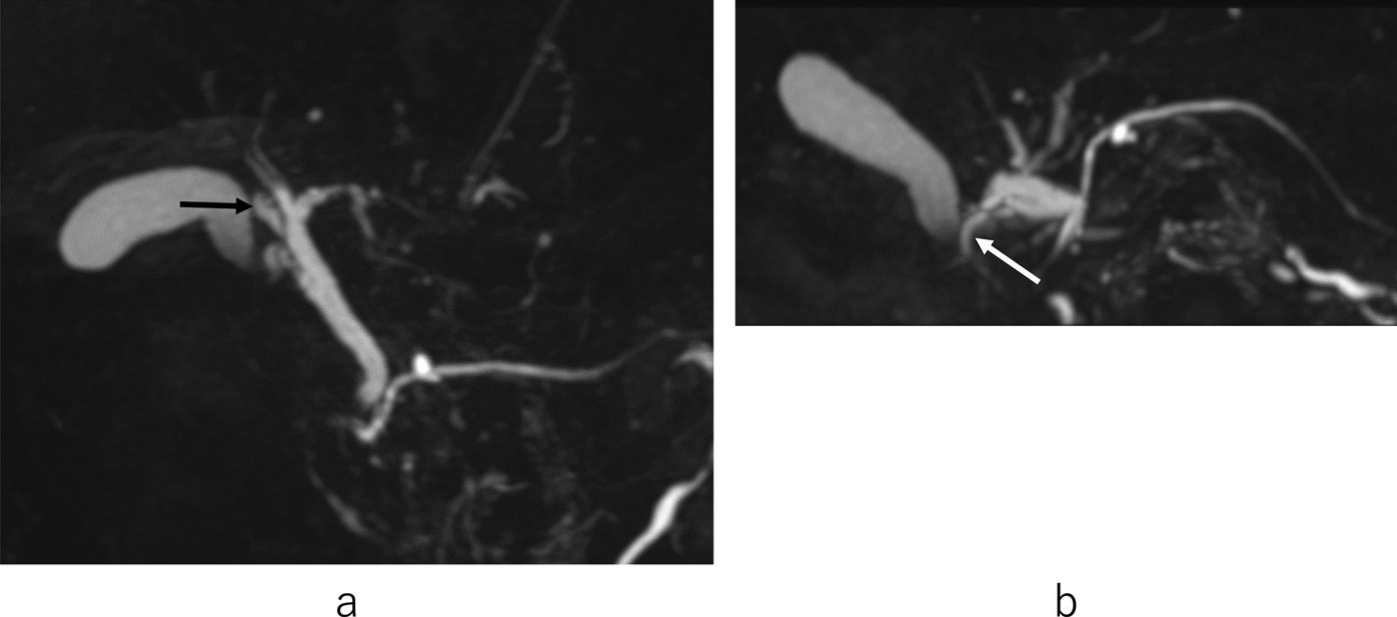
Anteroposterior view (A) and caudal view (B) of MRCP in case 2 show ARPHD draining into the extrahepatic bile duct (arrow). ARPHD, aberrant right posterior hepatic duct; MRCP, magnetic resonance cholangiopancreatography.

**Fig. 5:**
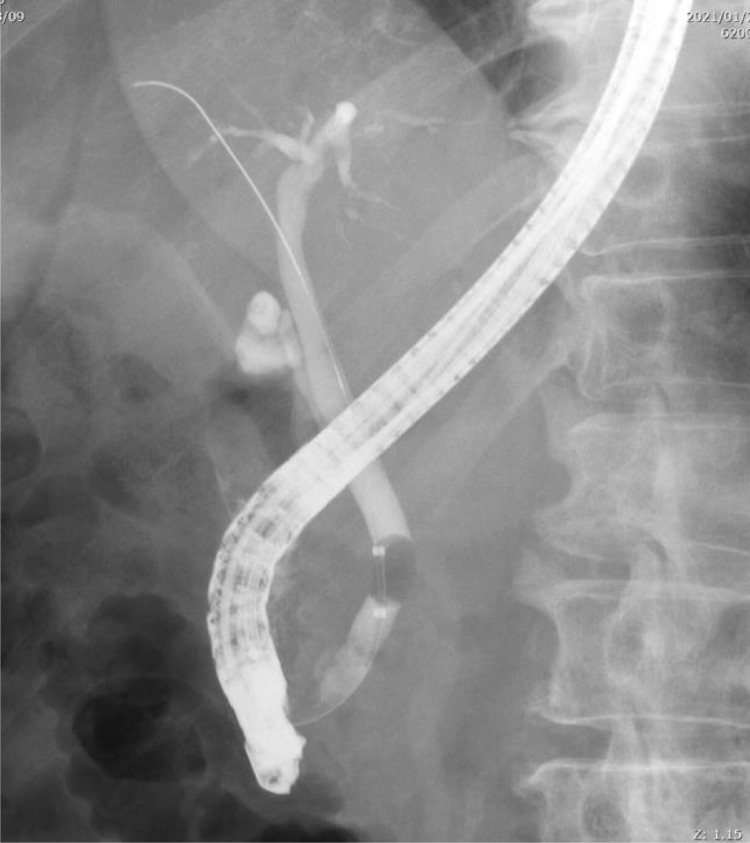
Endoscopic retrograde cholangiography 1 year ago in case 2 shows no ARPHD. ARPHD, aberrant right posterior hepatic duct.

**Fig. 6:**
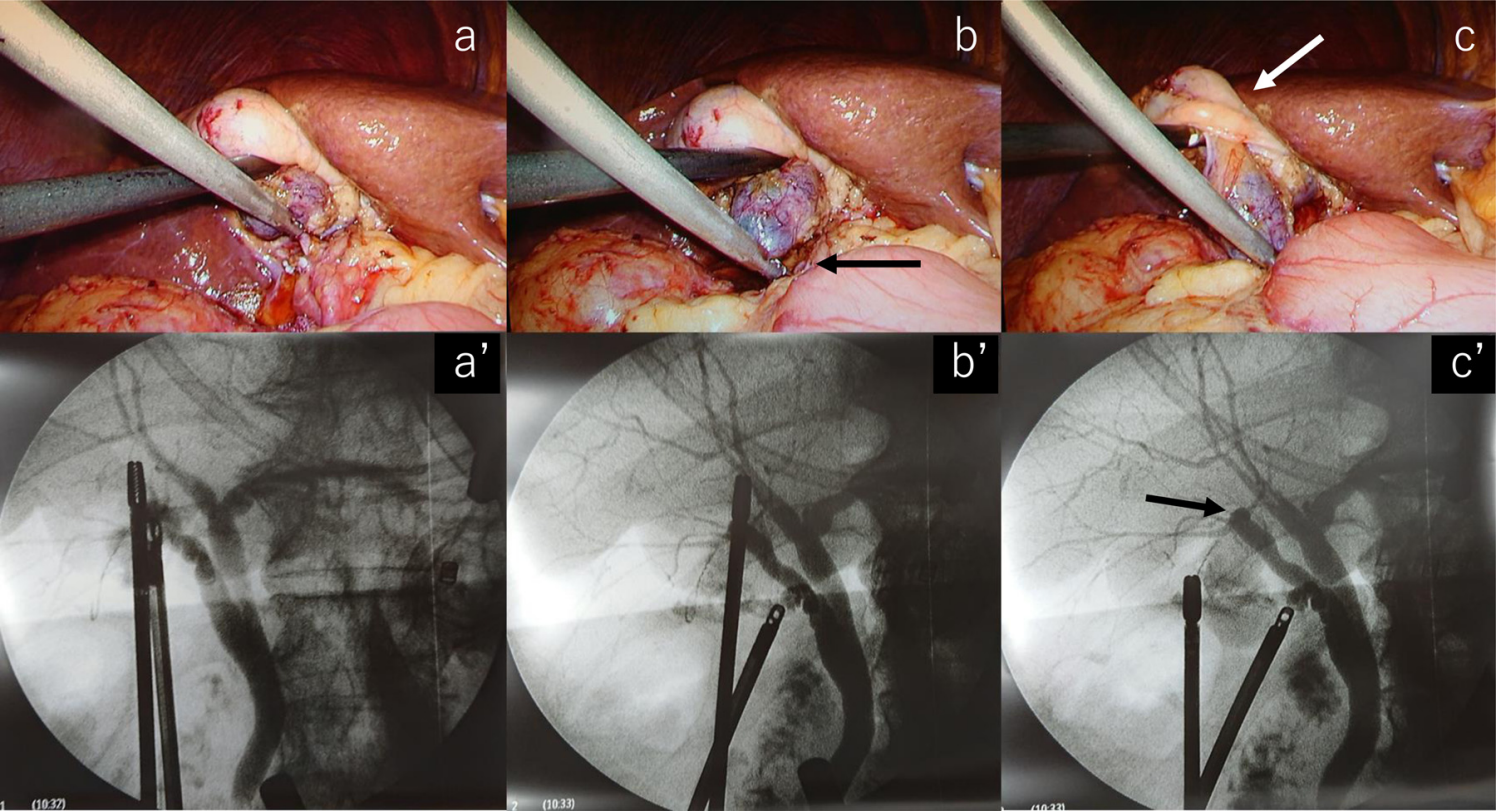
Dynamic IOC in case 2: Laparoscopic view (A) and anteroposterior view of cholangiogram (A’) show that the ARPHD overlaps with the cystic duct and is unclear. After pulling the cholangiograsper (black arrow) to the caudal side (B), the left anterior oblique view of cholangiogram (B’) shows the presence of ARPHD in addition to the cystic duct, but the peripheral bile ducts overlap with grasping forceps and are not clear. After pulling the grasping forceps (white arrow) to the outside (C), the left anterior oblique view of cholangiogram (C’) shows that the ARPHD (black arrow) is visualized throughout the peripheral bile ducts. There is no injury to ARPHD and no residual stone. ARPHD, aberrant right posterior hepatic duct; IOC, intraoperative cholangiography.

MRCP showed ARPHD draining into the extrahepatic bile duct (Fig. 4A, B), although ARPHD was not visualized by endoscopic retrograde cholangiography at the time of EST (Fig. 5). A 4-port LC was performed. Laparoscopy revealed a normal-looking gallbladder. A posterior infundibular approach was performed and was dissected along with the ss-i layer of the gallbladder [16,17]. Complete circumferential dissection of the infundibulum was achieved. After further dissection toward the cystic duct, the dissection between the cystic duct and cystic artery allowed clear visualization of the cystic duct. The cystic artery was divided after one clipping. The gallbladder neck was clipped. A small incision was made at the neck and a cannula for cholangiography was inserted. An anteroposterior view of cholangiography showed that the ARPHD overlapped with the cystic duct and was unclear (Fig. 6A, A’). After pulling the cholangiograsper to the caudal side, a left anterior oblique view of cholangiography showed the presence of ARPHD in addition to the cystic duct, however, the peripheral bile ducts were overlapped by grasping forceps and were not clear (Fig. 6B, B’). After pulling the grasping forceps to the outside, a left anterior oblique view of cholangiography showed ARPHD throughout the peripheral bile ducts (Fig. 6C, C’). After confirming that there was no injury to ARPHD and no residual stone by dynamic IOC, the cystic duct was doubly clipped and separated. Then the attached gallbladder was dissected from the liver bed and was extracted. The patient's postoperative course was uneventful. She was discharged 3 days after LC.

## Discussion

We reported a modified and dynamic IOC procedure that can identify ARPHD safely during LC. The modified IOC provided direct evidence of no injury to ARPHD.

The rate of bile duct injury during LC has not decreased despite advances in endoscopic surgery. Bile duct injury is sometimes severe and fatal [6], [7], [8], [9]. Misidentification of the cystic duct, severe inflammation, and bile duct anomalies have been reported as causes of bile duct injury. ARPHD, which is sometimes encountered, is a representative of bile duct anomalies. It has been reported that the prevalence of ARPHD is 2%-7% [1], [2], [3], [4], [5]. There are 5 types of ARPHD depending on the confluence of ARPHD, cystic duct, and common hepatic duct [1,^4^,5]. Many reports have warned of bile duct injury associated with ARPHD [6], [7], [8], [9].

Preoperative recognition of ARPHD is important to lessen the post-operative complications and injury to the bile duct. MRCP or DIC-CT is a non-invasive imaging modality that is useful in evaluating the anatomical variations of the bile duct system [10]. Preoperative MRCP is required at our hospital, and we sometimes encounter ARPHD. However, preoperative and intraoperative diagnoses of ARPHD are separate issues. The performance of the ss-i layer-keeping technique [5,16] is the basis of avoiding ARPHD injury. However, it provides only indirect evidence of no injury to ARPHD injury. The modified IOC provides direct evidence of no injury to ARPHD. Some authors questioned the usefulness of classic IOC to prevent ARPHD injury [4,5]. It is because if a catheter for a cholangiogram is intubated on the downstream side of the confluence of the ARPHD and cystic duct, reconstruction of the biliary tract is required in most cases [5]. The classic IOC procedure is based on an incision of the cystic duct; misidentification of it leads to bile duct injury. Classic IOC procedure that does not lead to bile duct injury avoidance needs to be modified. In modified IOC, cannulation is performed from the infundibulum or neck of the gallbladder. So modified IOC can avoid the ARPHD injury. If we aim to zero bile duct injury during LC; a modified IOC confirmation test is mandatory to avoid the bile duct injury as in the case of aircraft accident countermeasures [18]. So, we should not hesitate to perform modified IOC as well as MRCP.

In addition to the infundibulum cannulation method, modified IOC methods include the infundibulum puncture method, and IOC using endoscopic nasobiliary drainage tube [4] or percutaneous transhepatic gallbladder drainage tube. The modified IOC allows us to see where we are and what we are trying to separate. We disagree with the description of the Tokyo guideline for perioperative imaging *“Although there is no evidence for the value of IOC, preoperative MRCP, intraoperative fluorescence cholangiography, and intraoperative ultrasound may reduce bile duct injury.”* [19] . The former sentence and the latter sentence cannot be interpreted in the same dimension. This text is unfair in comparing the IOC with other methods. This sentence is misunderstood that the IOC is unnecessary. A prospective randomized study for modified IOC is not easy due to the low incidence of bile duct injury. Halawani HM et al [20]. reported that readmissions related to biliary complications following cholecystectomy were 1.61 times more likely in patients who underwent LC without cholangiography. Therefore, we support the strategies for minimizing bile duct injuries by the Society of American Gastrointestinal and Endoscopic Surgeons (SAGES). *“1. Use the critical view of safety method, 2. Understand the potential for aberrant anatomy, 3. Make liberal use of cholangiography or other methods.”* [21,22].

IOC records can be used to demonstrate the steps surgeons have taken to ensure surgical safety. We recommend the use of modified IOC instead of the classic IOC to achieve zero bile duct injury in LC. However, if the surgeon or operating room personnel are unfamiliar with the IOC procedures, they are not able to get good pictures and can take a significantly longer time to complete the procedure. This fact indicates that there may be a need for frequent educational sessions about the use of IOC.

We conclude that the modified and dynamic IOC can identify ARPHD safely and precisely during LC.

## Patient consent

Written informed consent was obtained from the patient for publication of this case report and accompanying images.
